# Childhood bullying victimization, emotion regulation, rumination, distress tolerance, and depressive symptoms: A cross‐national examination among young adults in seven countries

**DOI:** 10.1002/ab.22111

**Published:** 2023-09-08

**Authors:** Madelyn H. Labella, Neelamberi D. Klein, Georgina Yeboah, Claire Bailey, Ashley N. Doane, Debra Kaminer, Adrian J. Bravo, Cross-Cultural Addictions Study Team

**Affiliations:** 1 Department of Psychological Sciences, William & Mary, Williamsburg, Virginia, USA; 2 Department of Psychology, Old Dominion University, Norfolk, Virginia, USA; 3 Department of Psychology, University of Cape Town, Cape Town, South Africa

**Keywords:** adverse childhood experiences, bullying, depression, distress tolerance, emotion regulation, rumination

## Abstract

Existing research suggests a robust association between childhood bullying victimization and depressive symptoms in adulthood, but less is known about potential mediators of this link. Furthermore, there is limited cross‐national research evaluating similarities and differences in bullying victimization and its associations with mental health. The current study addressed gaps in the literature by evaluating cognitive and affective responses to stress (i.e., emotion regulation, rumination, and distress tolerance) as potential mediators of the link between recalled bullying victimization and current depressive symptoms among 5909 (70.6% female) college students from seven countries. Results revealed specific indirect associations of bullying victimization through distress tolerance and three out of four facets of rumination, as well as a persistent direct association of childhood bullying on adulthood depression. Emotion regulation strategies were not significantly associated with bullying victimization and did not mediate its association with depressive symptoms. Constrained multigroup models indicated that results were invariant across country and gender. Findings provide evidence of statistical mediation in a cross‐sectional sample and await replication in prospective studies. Rumination and distress tolerance may be promising targets for resilience‐promoting interventions among children experiencing peer victimization. Ongoing research is needed to clarify cross‐national patterns in childhood bullying, identify additional mediators accounting for the remaining direct association, and evaluate emotion regulation as a potential moderator of associations between bullying victimization and adult mental health.

## 1 | INTRODUCTION

Although definitions vary, bullying is often described as a chronic pattern of intentional aggression characterized by a power imbalance between the victim and the perpetrator (Felix et al., 2011). Bullying victimization is highly prevalent among youth, with a recent meta‐analysis yielding a weighted prevalence of 36.0% (Modecki et al., 2014). Accumulating evidence links experiences of bullying in childhood and adolescence with a variety of mental health problems that extend into late adolescence and adulthood (Arseneault et al., 2010; Evans‐Lacko et al., 2017). Of particular focus in the present study is the association between childhood bullying victimization and depression in adulthood. Meta‐analytic findings suggest that school bullying victimization is a significant risk factor for later depression (including up to 36 years later), even after accounting for numerous childhood risk factors (Ttofi et al., 2011). Another meta‐analysis found that children and adolescents who experience bullying victimization are twice as likely as nonvictims to develop depression, and concluded that there is convincing evidence from prospective studies for a temporal and likely causal relationship between bullying victimization and mental health outcomes like depression (Moore et al., 2017). The established link between childhood bullying victimization and adverse mental health outcomes, including depressive symptoms, has led to calls for “research on explanatory mechanisms involved in the development of mental health problems in bullied youths” (Arseneault et al., 2010, p. 726).

Existing research on mediators linking bullying victimization with depression has identified decrements in self‐esteem (Zhong et al., 2021) and resilient coping (Zhao et al., 2020; Zhou et al., 2017) as potential mechanisms for this association. However, more research is needed to incorporate well‐established emotional and cognitive risk factors for depression and to test how explanatory models function across national and cultural groups. The present study focuses on cognitive and affective responses to stress (i.e., emotion regulation, rumination, and distress tolerance) as potential mechanisms. Each of these constructs is robustly linked with depression (emotion regulation: Hu et al., 2014; Visted et al., 2018; rumination: Olatunji et al., 2013; Zhou et al., 2020; distress tolerance: Lass & Winer, 2020, Williams et al., 2013), and based on theory and prior research, represent strong candidates for mediating the relationship between bullying victimization and depression.

### 1.1 | Emotion regulation

Emotion regulation refers to the processes by which individuals modify components of an emotional response—for example, decreasing the duration and intensity of negative emotions like sadness, anger, and shame—to facilitate adaptive functioning (Peña‐Sarrionandia et al., 2015). According to the developmental psychopathology framework, childhood adversity may disrupt the development of effective emotion regulation skills through repeated experiences of strong negative emotions and chronic activation of the stress response system (Pollak, 2015). This is particularly concerning because emotion regulation difficulties have been implicated in a broad range of psychopathology, including depressive disorders (Joormann & Stanton, 2016). Consistent with theory, research has repeatedly identified emotion regulation as a key mediating factor between adverse childhood experiences and later mental health outcomes (Cloitre et al., 2019; Miu et al., 2022). For example, in a clinical sample of German adults with Major Depressive Disorder, deficits in general emotion regulation skills mediated the relationship between childhood trauma and both depression severity and depression lifetime persistency (Hopfinger et al., 2016). When examining mediation by specific emotion regulation strategies, Huh et al. (2017) found that both adaptive (e.g., cognitive reappraisal) and maladaptive (e.g., expressive suppression) emotion regulation strategies mediated the relationship between childhood trauma and depressive symptom severity among South Korean adults hospitalized for depressive disorders.

However, little research examines the potential mediating role of emotion regulation for effects of bullying victimization. However, childhood bullying, as with other early adversity, may evoke strong emotional reactions that need to be regulated and may disrupt the development of effective emotion regulation skills over time. Additionally, little is known about cross‐national differences in mediation by emotion regulation. This is a particularly important question given known cultural differences in normative emotion regulation strategies. For example, cross‐national studies have found that individuals from cultures emphasizing social order tend to use suppression more often than those from individualistic cultures (Matsumoto et al., 2008). Importantly, converging research suggests that the negative consequences of expressive suppression may be mitigated—or even reversed—based on cultural norms (Arens et al., 2013; Butler et al., 2007, 2009; Schunk et al., 2022). Taken together, this suggests that the role emotion regulation plays in the relationship between early adversity and depression could be culture‐specific, highlighting the value of cross‐national research.

### 1.2 | Rumination

Rumination is often defined as repetitive thinking about causes, consequences, and symptoms of negative affect and mood states, and is a well‐established cognitive risk factor for depression (Nolen‐Hoeksema et al., 2008). Rumination has been conceptualized both as an involuntary stress response (Malamut & Salmivalli, 2021) and a discrete emotion regulation strategy (Miu et al., 2022). In either case, rumination evoked by experiences of victimization offers a potential pathway by which bullying confers risk for later adjustment problems. Consistent with this proposal, a meta‐analysis of longitudinal studies documented bidirectional associations linking bullying victimization with rumination and related internalizing problems over time (Reijntjes et al., 2010).

A wealth of evidence shows rumination playing a mediating role in the association between bullying victimization and depressive symptoms across a variety of demographic groups, including American middle schoolers (Dorio et al., 2019; Mathieson et al., 2014), Finnish schoolchildren (Peets et al., 2021), Chinese junior high students (Chu et al., 2019; Liu et al., 2020), and Italian adolescents (Gini et al., 2019). Related research has identified indirect associations of victimization through rumination on other maladaptive outcomes, including relational aggression (Li et al., 2021) and bullying perpetration (Malamut & Salmivalli, 2021). Overall, results indicate that bullying victimization is strongly related to rumination and that rumination may explain, at least in part, the relationship between victimization and depressive symptomology.

Although this has been replicated in multiple cultural groups, little research has examined whether the strength and significance of these relationships may vary cross‐nationally. Some studies suggest that rumination is both more common and less strongly associated with psychological maladjustment in collectivistic versus individualistic cultures, perhaps because of higher value placed on prosocial negative emotions as well as greater tendency to self‐distance from one’s emotions (Chang et al., 2010; De Vaus et al., 2018; Schunk et al., 2022). However, a recent cross‐national study found that culture did not moderate links between rumination and symptoms of depression and posttraumatic stress (Li et al., 2022). More research is needed regarding potential differences in pathways linking victimization, rumination, and depression based on cultural values and national origin.

### 1.3 | Distress tolerance

Distress tolerance has been defined as the ability to persist in goal‐directed activity when experiencing psychological distress (Brown et al., 2002) or the capacity to withstand negative internal states without seeking to avoid or escape them (Simons & Gaher, 2005). Research suggests that lower distress tolerance may be associated with an increased likelihood and severity of depression (Cummings et al., 2013; Ellis et al., 2013; Williams et al., 2013) and that the degree to which stressful experiences increase risk for depression depends on the capacity to tolerate distress (Lass & Winer, 2020). Evidence regarding the link between early adversity and distress tolerance is mixed: Berenz et al. (2018a, 2018b) found that childhood physical abuse and emotional neglect predicted higher perceived distress tolerance, whereas childhood emotional abuse and family violence predicted lower behavioral distress tolerance (i.e., faster desistance on an unpleasant task). Relatedly, cumulative violence exposure (both family and community violence) was associated with lower behavioral distress tolerance in a sample of urban adolescents (Heleniak et al., 2021). Based on these results, we anticipate that chronic victimization by peers may interfere with the developing capacity to tolerate distress (similar to effects of emotional abuse and witnessing violence), and that lower distress tolerance will confer risk for mood disturbance.

Little is known about cross‐national differences in distress tolerance and its associations with psychological outcomes. However, within the United States, race/ethnicity has been shown to moderate associations between distress tolerance and psychiatric symptoms in early and late adolescence (Daughters et al., 2009, 2013), and a recent systematic review highlighted stronger links between distress tolerance and smoking dependence in Iranian versus Western samples (Veilleux, 2019). In combination with theoretical considerations regarding cultural norms for experiencing and expressing negative emotion, these findings highlight a need for cross‐national comparison of pathways linking stressful life experiences, distress tolerance, and depression.

### 1.4 | Present study

Recent literature has called for an examination of cognitive and affective variables as potential mechanisms linking childhood bullying victimization and mental health problems (see Arseneault et al., 2010). To this end, the present study aimed to examine emotion regulation strategies (i.e., reappraisal and suppression), rumination (assessed in a multifaceted approach), and distress tolerance as potential mediators of the relationship between childhood bullying victimization and depressive symptoms in a convenience sample of young adults from seven countries (United States, Canada, Spain, England, Argentina, Uruguay, and South Africa). Based on prior research and theory, we expected that endorsement of having experienced childhood bullying victimization would be associated with greater reports of adulthood depressive symptoms via lower use of reappraisal strategies, greater use of suppression strategies, higher reports of ruminative thinking, and lower distress tolerance. Bullying victimization is not a culturally specific phenomenon but rather widely prevalent globally (Cook et al., 2010; Elgar et al., 2015; Eslea et al., 2004); however, cross‐national studies comparing the associations of bullying victimization on mental health outcomes are limited. To this end, we also explored whether findings were culturally universal or culturally specific by testing the structural invariance of the model across both countries and gender.

## 2 | METHOD

### 2.1 | Participants and procedures

Participants were college students (*n* = 9171) conveniently recruited from 12 universities spanning seven countries (United States, Canada, Spain, England, Argentina, Uruguay, and South Africa; countries were selected based on prior relationships among the investigators) to complete an online survey exploring risk and protective factors of substance use and addictive behaviors (see Bravo et al., 2021, for more information). To minimize participant burden, we used a planned missingness design (i.e., matrix sampling; Graham et al., 2006; Schafer, 1997) such that participants first completed demographic and substance use measures followed by a random selection of 12 measures from a larger pool (17 total measures). Due to our missing‐data‐by‐design procedure, the analytic sample for the present study was 5909 (70.6% female) students who completed the measure of Adverse Childhood Experiences measure (64% of total sample) as well as the Inventory of Depression and Anxiety Symptoms (completed by every student; United States, *n* = 2900, 67.4% female; Canada, *n* = 1155, 66.2% female; South Africa, *n* = 463, 83.3% female; Spain, *n* = 469, 70.9% female; Uruguay *n* = 89, 91.0% female; Argentina, *n* = 514, 76.5% female; England, *n* = 319, 81.3% female). Study procedures were approved by the institutional review boards (or the international equivalent) for each participating university.

### 2.2 | Measures

#### 2.2.1 | Childhood bullying victimization

Experience of childhood bullying victimization was assessed using the Adverse Childhood Experiences‐International Questionnaire (ACE‐IQ), which was created for use as a cross‐national scale of adverse childhood experiences (World Health Organization, 2018). A Spanish version was developed by the research team and used at Spanish‐speaking sites (see Kaminer et al., 2023 for further details). The ACE‐IQ assesses exposure to 13 ACEs; however, for the present study we focused on experiences of childhood bullying. Bullying was defined as when a young person or group of young people say or do bad and unpleasant things to another young person, and also when a young person is teased a lot in an unpleasant way or when a young person is left out of things on purpose (World Health Organization, 2018). Participants were first asked how often they were bullied during the first 18 years of life, to which the response was either many times, a few times, once or never. Participants who responded “never” did not receive the second item, asking them to indicate which forms of bullying they experiences (e.g., “I was hit, kicked, pushed, shoved around, or locked indoors;” for rates of endorsement for specific bullying experiences across countries, see Table 1). The present study employed the binary scoring method (World Health Organization, 2018), in which any level of exposure to bullying victimization (whether once or multiple experiences) was coded as having experienced childhood bullying victimization (i.e., 0 = no experience, 1 = experienced any childhood bullying victimization).

#### 2.2.2 | Emotion regulation

Emotion regulation strategies were assessed using the 10‐item Emotion Regulation Questionnaire (ERQ; Gross & John, 2003) and its corresponding Spanish version (Cabello et al., 2013) for Spanish‐speaking students. This scale uses six items to measure individual differences in cognitive reappraisal (*α* = .89 total sample; .82 < *α* > .90 across countries) and four items to measure expression suppression (*α* = .78 total sample; .73 < *α* > .80 across countries) (Gross & John, 2003). Participants responded to items on a 7‐point Likert scale (1 = *Strongly disagree* to 7 = *Strongly agree*).

#### 2.2.3 | Rumination

Rumination was assessed using the 15‐item version (Tanner et al., 2013) of the Ruminative Thoughts Style Questionnaire (RTSQ; Brinker & Dozois, 2009) and its Spanish version (Bravo et al., 2019) for Spanish‐speaking students. The RTSQ measures general tendencies to engage in ruminative behaviors on a 7‐point Likert scale (1 = *Very untrue of me* and 7 = *Describes me very well*). The scale is composed of four subscales (Tanner et al., 2013): problem‐focused thoughts (i.e., limited problem‐solving capabilities and inefficient information processing) (*α* = .90 total sample; .84 < *α* > .90 across countries), counterfactual thinking (i.e., focus on alternative outcomes) (*α* = .89 total sample; .83 < *α* > .90 across countries), repetitive thoughts (i.e., intrusive, persistent, and automatic thoughts) (*α* = .94 total sample; .92 < *α* > .96 across countries), and anticipatory thoughts (i.e., persistent future‐oriented thoughts) (*α* = .77 total sample; .69 < *α* > .80 across countries).

#### 2.2.4 | Distress tolerance

Distress tolerance was assessed using the 15‐item Distress Tolerance Scale (DTS; Simons & Gaher, 2005) and its Spanish version (Sandín et al., 2017) for Spanish‐speaking students. The DTS assesses participants’ ability to withstand negative psychological states (*α* = .93 total sample; .90 < *α* > .94 across countries) on a 5‐point Likert scale (1 = *Strongly agree* to 5 = *Strongly disagree*).

#### 2.2.5 | Inventory of depression and anxiety

Depressive symptoms were assessed using the Inventory of Depression and Anxiety Symptoms (IDAS; Watson et al., 2007). A Spanish version was created by the research team and used at Spanish‐speaking sites (see Kaminer et al., 2023 for further details). The IDAS was created to complement existing measures of depression and anxiety and is composed of several specific symptom scales and two broader scales (general depression and dysphoria). The 20‐item general depression scale was developed to reflect the range of symptoms associated with major depressive disorder, and includes items from scales tapping dysphoria, suicidality, lassitude, insomnia, appetite loss, and well‐being (reverse‐keyed). Participants recorded how much they experienced each item during the past two weeks using a 5‐point Likert Scale (1 = *not at all*, 5 = *extremely*). After seven items were dropped due to high intercorrelations causing misfit, the resulting 13‐item version of the depression subscale was found to be invariant across countries (*α* = .90 total sample; .86 < *α* > .91 across countries).

#### 2.2.6 | Measurement invariance

Importantly, invariance testing of all appropriate measures demonstrated metric invariance across the countries, which is necessary when examining associations between study constructs across different groups (see Supporting Information: Table 1 for results of all cross‐national invariance testing). For all constructs, items were averaged or summed such that higher scores indicate higher endorsement of that construct. Internal consistency (i.e., alphas) of all appropriate study variables for each country are presented in Supporting Information: Table 2.

### 2.3 | Data analyses plan

The study aims were tested using a comprehensive mediation model conducted using M*Plus 8.3* (Muthén & Muthén, 1998–2017). Specifically, a single fully saturated path analysis model was run in which experience of childhood bullying victimization was examined as a statistical predictor of depressive symptoms via rumination facets, emotion regulation strategies, and distress tolerance (see Figure 1). Within the model, we simultaneously estimated paths of all mediators to examine unique indirect associations for emotion regulation strategies of reappraisal and suppression, each subcomponent of rumination, and total scores on distress tolerance. Age and socioeconomic status were included as predictors (i.e., covariates) of all variables in the model. We examined the total, direct, and indirect associations of each predictor variable on depressive symptoms using bias‐corrected bootstrapped estimates based on 10,000 bootstrapped samples. Statistical significance was determined by 99% bias‐corrected bootstrapped confidence intervals that do not contain zero.

To test for structural invariance of the model (i.e., whether country or gender moderates specific pathways), we conducted *χ*^2^ difference tests (*p* < .01) comparing an unconstrained model, in which regression paths were free to vary across country and gender (separate models), to a constrained model, in which corresponding regression paths were forced to be equivalent across countries and genders. Given that the χ^2^ test statistic is sensitive to sample size (Brown, 2015), we also used a model comparison criterion of ΔCFI ≥ 0.01 (Cheung & Rensvold, 2002) to indicate significant decrement in fit when testing for structural invariance. All data and analytic outputs are available at 10.17605/OSF.IO/4JD2C.

## 3 | RESULTS

Overall, 56.1% of our total sample reported at least one experience of childhood bullying victimization. By country, Uruguay had the highest proportion of participants report some experience of bullying victimization (71.9%) while Spain had the lowest (40.1%). The most common form of bullying was being “made fun of because of how my body or face looked” (52.9%), followed by being “left out of activities on purpose or completely ignored” (45.8%). Rates of endorsement of bullying victimization across specific experiences across countries are presented in Table 1. Appearance‐based bullying was also the most common form of bullying endorsed in every country except in Canada and England, which showed higher rates of participants being purposefully excluded from activities (50.7% and 58.4%, respectively). Physical bullying (i.e., “hit, kicked, pushed, shoved around, or locked indoors”) had the lowest endorsement overall (8.6%) and in every country in our sample except England, where “bullying on the basis of race, nationality, or colour” was the least common (6.9%).

Bivariate correlations and descriptive statistics of all study variables are presented in Table 2. Broadly, childhood bullying showed expected bivariate associations with rumination and distress tolerance, but not emotion regulation. Depression was significantly associated with bullying victimization and all candidate mediators. Further, exploratory tests for gender differences revealed significant mean differences (at 99% confidence intervals) on every continuous variable; however, the effect sizes for all of these comparisons were quite small (0.11 > Cohen’s *d* values < 0.32 for all comparisons). Regarding bullying victimization, females (58.2%) reported experiencing bullying victimization more compared to males (50.2%). Results of these exploratory analyses are available at 10.17605/OSF.IO/4JD2C.

### 3.1 | Mediation model results

The total, indirect, and direct associations for the mediation model are summarized in Table 3 (indirect associations) and Figure 1 (direct associations). Constrained multigroup models (gender model: *χ*^2^[15] = 14.66, CFI = 1.00; country model: *χ*^2^[75] = 112.31, CFI = 0.997) compared to the freely estimated model (fully saturated with CFI = 1.00 and *χ*^2^ = 0.00 for each model) indicated model invariance across countries (Δ*χ*^2^[75] = 112.31, *p* = .003, ΔCFI = −0.003) and gender (Δ*χ*^2^[15] = 14.662, *p* = .476, ΔCFI = 0.00). Given evidence of invariance, we present results of our model within the total sample.

Within our mediation model, we found a significant total association (*β* = .21, 99% confidence interval [CI]: [0.18, 0.24]) and total indirect association (*β* = .11, 99% CI: [0.08, 0.13]) of childhood bullying on depressive symptoms. In examining specific indirect paths, we found that problem‐focused thoughts, repetitive thoughts, anticipatory thoughts, and distress tolerance—but not cognitive reappraisal, expressive suppression, or counterfactual thinking—uniquely mediated the association of experiencing childhood bullying victimization on depressive symptoms (see Table 3). Specifically, experiencing childhood bullying victimization was associated with higher reports of adulthood depressive symptoms via higher problem‐focused thoughts, repetitive thoughts, and anticipatory thoughts, as well as lower distress tolerance. These mediators uniquely accounted for 13.53% (problem‐focused thoughts), 15.46% (repetitive thoughts), 10.14% (anticipatory thoughts), and 11.11% (distress tolerance) of the total variance shared between bullying victimization and depressive symptoms. Above and beyond the paths of our mediators, we found a significant direct association of experiencing childhood bullying victimization on depressive symptoms during young adulthood (*β* = .101, 99% CI: [0.07, 0.13]).

## 4 | DISCUSSION

The present study examined links between bullying victimization and depressive symptoms via ruminative thought styles, emotion regulation strategies, and distress tolerance and further examined the invariance of associations across nationality and gender. Within our comprehensive model, we found that childhood bullying victimization had significant indirect associations on depressive symptoms in adulthood through three aspects of ruminative thought as well as overall distress tolerance. However, effects of bullying victimization were not uniquely mediated by a fourth aspect of rumination (i.e., counterfactual thinking) or emotion regulation strategies. Additionally, bullying victimization had a significant direct association on depressive symptoms accounting for all candidate mediators and covariates. Notably, our final model was structurally invariant across nationality and gender, indicating these statistical relationships were consistent across participants from seven countries and two gender identities.

Overall, results corroborated previous research documenting positive associations between childhood bullying victimization and depressive symptoms in young adulthood (e.g., Ttofi et al., 2011), as well as links between depression and emotion regulation (Hu et al., 2014; Visted et al., 2018), rumination (Olatunji et al., 2013; Zhou et al., 2020), and distress tolerance (Lass & Winer, 2020, Williams et al., 2013). Analyses also supported prior theory and research identifying rumination as a key mediator between bullying victimization in childhood and depression in adulthood (Dorio et al., 2019; Malamut & Salmivalli, 2021; Mathieson et al., 2014; Peets et al., 2021). In the current study, significant indirect paths were detected for three aspects of rumination: problem-focused thoughts, repetitive thoughts, and anticipatory thoughts. These results are line with the Response Styles Theory of depression (RST; Nolen‐Hoeksema & Morrow, 1991; Nolen‐Hoeksema, 1991), which proposes that a ruminative response style in response to a traumatic stressor (in this case, bullying victimization) prolongs and intensifies depressed moods that might otherwise diminish more rapidly. Feelings of hopelessness induced by ruminative thinking may partly explain why rumination in response to a stressor increases the risk for depression over time (Sarin et al., 2005). The current study provides preliminary support for this theoretical explanation by documenting statistical mediation by rumination in a cross‐sectional sample. Of note, counterfactual thinking was not a significant mediator in the current study, in contrast to other aspects of rumination. This may reflect high covariation among subscales of the Ruminative Thought Styles Questionnaire and/or indicate that other facets of rumination are especially relevant to the link between bullying victimization and depression. Repetitive thinking about interpersonal problems and their potential recurrence may confer greater risk for mood disturbance than habitually thinking about desired alternatives to bullying.

Unexpectedly, neither cognitive reappraisal nor expression suppression significantly mediated the association of bullying victimization on depressive symptoms. This is surprising given abundant evidence that disrupted emotion regulation (including alteration in strategy use) mediates effects of other early adversities on depressive symptoms in adulthood (Cloitre et al., 2019; Huh et al., 2017; Miu et al., 2022). Because children’s emerging emotion regulation skills are socialized primarily by family members (Morris et al., 2007), adverse childhood experiences occurring within the family, such as child maltreatment, may be more deleterious for emotion‐regulatory development than extrafamilial adversities. Instead, emotion regulation strategies acquired primarily in the family environment may serve to *moderate* associations between childhood bullying on later mental health. Indeed, Garnefski and Kraaij (2014) found that maladaptive emotion regulation strategies strengthened the link between bullying victimization and internalizing symptoms, whereas adaptive strategies weakened this link, consistent with a buffering role. Future research should examine mediating and moderating roles of emotion regulation in the link between different types of early adversity and subsequent depression.

Regarding the final candidate mediator, the current study provides novel evidence that distress tolerance may mediate the link between bullying victimization and depressive symptoms. This is consistent with a body of research identifying lower distress tolerance as a key pathway from childhood maltreatment to mental health problems in adulthood (Robinson et al., 2021). Individuals reporting victimization by peers are thus at risk for lower capacity to tolerate distress, which in turn is associated with concurrent depression. Some previous research suggests that distress tolerance may influence depression through the mechanisms of perseverative thinking and/or maladaptive coping (Jeffries et al., 2016; McDermott et al., 2018), which overlap with other candidate mediators. However, our findings indicate a significant indirect path through distress tolerance even when accounting for rumination and emotion regulation, suggesting a unique role for the diminished capacity to tolerate distress.

Importantly, half the total effect of bullying victimization on depressive symptoms persisted controlling for all candidate mediators. This suggests that other mechanisms are involved in explaining persistent associations between childhood bullying and depressive symptoms in young adulthood. Future research should incorporate additional mediators—for example, decreased self‐esteem (Zhong et al., 2021) and/or ongoing interpersonal stressors in adolescence and young adulthood—as potential pathways from bullying to depression.

A final contribution of the current study involves a cross‐national comparison of prevalence rates of bullying. Prevalence rates have been difficult to compare across individual studies due in part to differences in methodology (e.g., definitions, measures, timeframe; Smith, 2016). One benefit of the present study is facilitation of direct comparison due to the consistency of a standardized measure. We found that the highest total rates of bullying victimization were reported in Uruguay, whereas the lowest prevalence were reported in Spain. Rates of physical bullying were consistently low (1.6%–10.0%), whereas appearance‐based bullying was fairly common across countries (49.1%–65.6%). Prevalence rates of bullying due to race, nationality, or color were generally highest in the North American countries, and the greatest cross‐national differences were observed for exclusion, which was twice as common in England as Uruguay. Future research is needed to examine bullying across nations and cultures to clarify similarities and differences in peer victimization experiences.

### 4.1 | Strengths and limitations

Strengths of the current study include its large sample size and simultaneous testing of multiple candidate mediators, which corroborated the unique mediating role of rumination and provided novel support for mediation through distress tolerance. Furthermore, this large‐scale international collaboration facilitated rigorous examination of structural invariance across countries. However, we did not take steps to ensure our samples across countries were representative; therefore, our generalizability is limited. Although limited, insights gained by comparing effects across countries elucidate potential areas for future research. Other limitations include exclusive reliance on self‐report measurement, which raises concerns about social desirability bias, retrospective recall of childhood experiences, and shared method variance. Additionally, the nonexperimental study design precludes causal inference. Because the study is cross‐sectional, we are unable to disentangle temporal direction of associations: although we have identified indirect paths through rumination and distress tolerance, our predictor, candidate mediators, and focal outcome were measured at the same time point. It is thus possible that depressive symptoms may precede increases in rumination and decreases in distress tolerance, or that psychological functioning in young adulthood shapes recollections and interpretations of childhood bullying. The findings do, however, suggest potential mediation candidates that can be explored in longitudinal prospective studies of the relationship between childhood bullying victimization and symptoms of depression in young adulthood. Such studies may provide support for treatments that target rumination, emotion regulation and distress tolerance among youth and young adults who have survived childhood bullying experiences.

## 5 | CONCLUSIONS

Overall, the current study represents a valuable step forward in delineating potential pathways from childhood bullying victimization to young adulthood depression. Across a large sample of college students from 12 universities in seven countries, rumination and distress tolerance (but not emotion regulation strategies) emerged as unique statistical mediators of the link between childhood bullying victimization and adulthood depression. Importantly, results were invariant across country and gender, suggesting high external validity. Findings await replication in prospective studies following individuals exposed to early adversity, including childhood bullying, over time. Results revealed several fruitful directions for future research, including cross‐cultural comparison of peer victimization experiences, examination of additional mediators (e.g., self‐esteem, interpersonal functioning in adolescence and young adulthood) in the link between bullying and depression, and evaluation of moderation by emotion regulation strategy use. This line of research has the potential to clarify key mechanisms and protective factors in the link between bullying victimization and mental health, illuminating potential targets for future resilience‐promoting interventions.

## Supplementary Material

Online Supplement

Additional supporting information can be found online in the Supporting Information section at the end of this article.

## Figures and Tables

**FI GURE 1:**
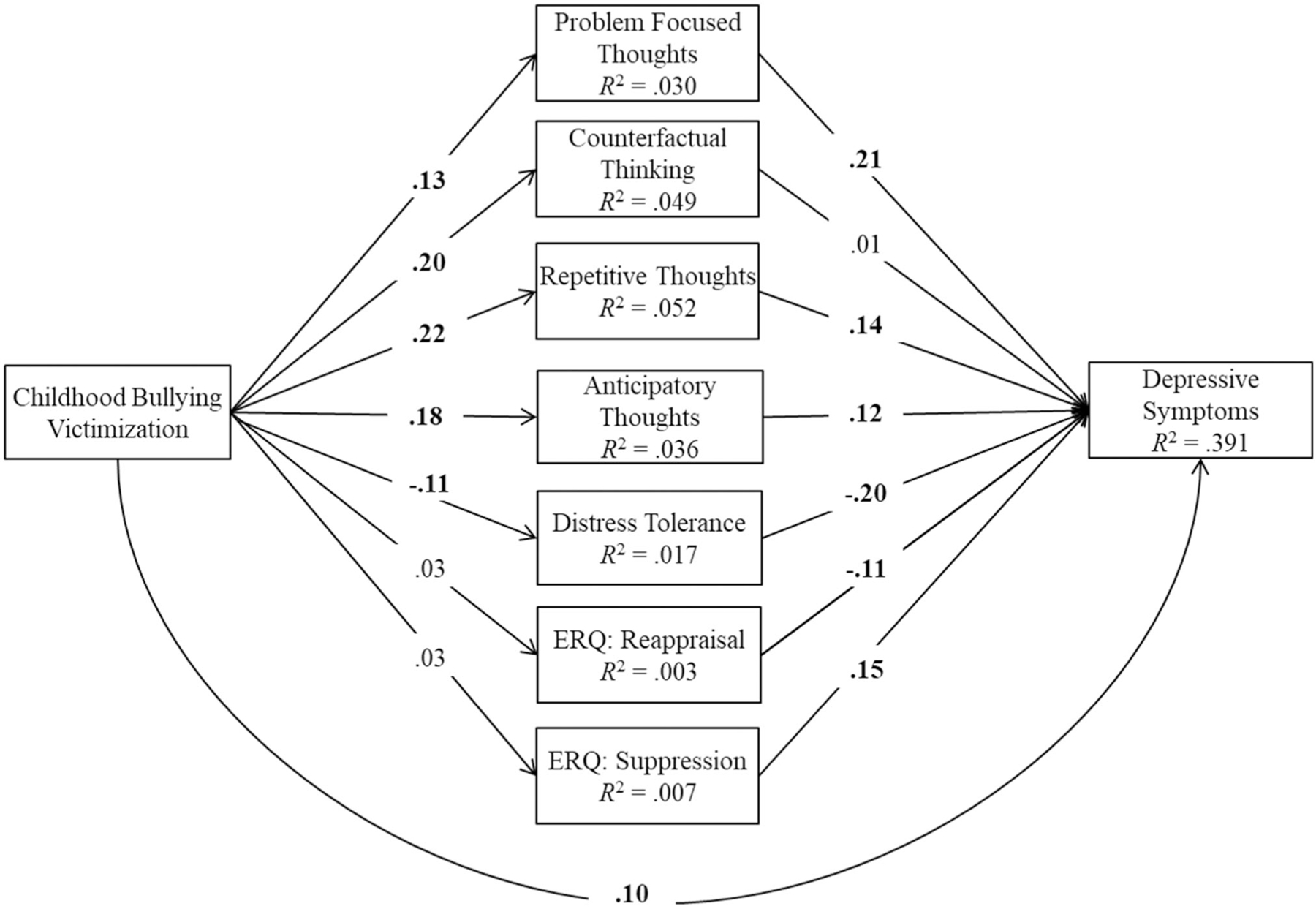
Standardized effects of mediation model in total sample. This figure shows the standardized effects of the mediation model. Significant associations are in bold typeface for emphasis and were determined by a 99% bias‐corrected standardized bootstrapped confidence interval (based on 10,000 bootstrapped samples) that do not contain zero. The disturbances among mediators were allowed to correlate and all effects accounted for age and SES as covariates. These effects are not shown in the figure for reasons of parsimony but are available at 10.17605/OSF.IO/4JD2C.

**TABLE 1:** Frequency of specific bullying experiences across sites.

	Total	USA	Canada	South Africa	Spain	Argentina	Uruguay	England
Total	56.1%	54.6%_a_	58.0%_a,b,c_	64.1%_b,c_	40.1%_d_	65.8%_c_	71.9%_b,c_	54.2%_a,b_
Type of bullying								
Hit, kicked, pushed, shoved around, or locked indoors	8.6%	8.4%_a_	10.0%_a_	8.1%_a_	6.9%_a_	8.6%_a_	1.6%_a_	9.8%_a_
I was made fun of because of my race, nationality, or color	17.6%	21.1%_a_	19.1%_a,b_	15.8%_a,b,c_	14.4%_a,b,c_	9.5%_c_	4.7%_b,c_	6.9%_c_
I was left out of activities on purpose or completely ignored	45.8%	47.3 _a,b_	50.7% _a,b_	41.1% _b,c_	42.6% _a,b,c_	32.2% _c_	23.4%_c_	58.4%_a_
I was made fun of because of how my body or face looked	52.9%	52.1%_a,b_	50.4%_b_	53.2%_a,b_	52.7%_a,b_	60.9%_a_	65.6%_a,b_	49.1%_a,b_
I was bullied in some other way	37.7%	38.3%_a,b,c,d_	40.6%_c,d_	42.1%_b,d_	34.0%_a,b,c,d_	30.2%_a_	26.6%_a,b,c,d_	37.0_a,b,c,d_

*Note*: Each subscript letter denotes a subset of country categories whose column proportions do not differ significantly from each other at the *p* < .05 level. Due to experimenter error, two items were presented as the same time (i.e., within the same question) and were excluded from these frequency analyses (I was made fun of because of my religion and I was made fun of with sexual jokes, comments, or gestures).

**TABLE 2:** Bivariate correlations among study variables in total sample (n = 5,910).

	1	2	3	4	5	6	7	8	9	10	11	*M*	SD
1. ACE ‐ Bullying	‐‐‐											0.56	0.50
2. Problem Focused Thoughts	.13	‐‐‐										3.70	1.39
3. Counterfactual Thinking	.20	.62	‐‐‐									4.75	1.48
4. Repetitive Thoughts	.22	.61	.75	‐‐‐								4.89	1.52
5. Anticipatory Thoughts	.18	.65	.63	.63	‐‐‐							4.35	1.49
6. Distress Tolerance	−.12	−.42	−.25	−.27	−.29	‐‐‐						3.16	0.83
7. ERQ ‐ Reappraisal	.03	−.09	.09	.07	.06	.20	‐‐‐					4.62	1.15
8. ERQ – Suppression	.03	.27	.22	.20	.19	−.11	.18	‐‐‐				3.92	1.30
9. IDAS Depression	.21	.53	.41	.45	.44	−.42	−.12	.27	‐‐‐			28.63	10.27
10. Age	.07	−.07	−.08	−.04	−.05	.03	.05	−.04	−.02	‐‐‐		20.23	3.97
11. Socioeconomic Status	−.08	−.07	−.04	−.03	−.03	.06	.00	−.06	−.11	−.17	‐‐	2.97	0.86

*Note*: Significant correlations are bolded for emphasis and were determined by a 99% bias‐corrected standardized bootstrapped confidence interval (based on 10,000 bootstrapped samples) that does not contain zero. Subjective socioeconomic status was assessed on a 5‐point scale: 1 = *Poor or just barely making it*, 2 = *Working or labor class*, 3 = *Middle class*, 4 = *Upper middle class*, 5 = *Wealthy*.

**TABLE 3:** Summary of total, indirect, and direct effects of childhood bullying on depressive symptoms in a comprehensive mediation model.

Outcome variables	Depressive symptoms	
Predictor variable: Experience of bullying	*β*	99% CI
Total	.207	0.18, 0.24
Total indirect^a^	.106	0.08, 0.13
Specific indirect		
Problem‐focused thoughts	.028	0.02, 0.24
Counterfactual thinking	.001	−0.01, 0.01
Repetitive thoughts	.032	0.02, 0.05
Anticipatory thoughts	.021	0.01, 0.03
Distress tolerance	.023	0.01, −0.03
ERQ—reappraisal	−.003	−0.01, 0.001
ERQ—suppression	.004	−0.002, 0.011
Direct	.101	0.07, 0.13

*Note*: Significant associations are in bold typeface for emphasis and were determined by a 99% bias‐corrected standardized bootstrapped confidence interval (based on 10,000 bootstrapped samples) that does not contain zero.

a Reflects the combined indirect associations within the model.

## Data Availability

The data that support the findings of this study are openly available in Project CABS— Bullying Paper at https://doi.org/10.17605/OSF.IO/4JD2C.
