# Supplementary material for: Childhood bullying victimization, emotion regulation, rumination, distress tolerance, and depressive symptoms: A cross‐national examination among young adults in seven countries
Source: Aggress Behav. Author manuscript; Available in PMC 2025 Jul 15. (PMC12261286; doi:10.1002/ab.22111)
Supplement: Online Supplement [file NIHMS2090716-supplement-Online_Supplement.docx]

Supplemental Table 1

*Measurement invariance testing results for study measures across countries using WLSMV estimator*

| *Emotion Regulation Questionnaire (2 factors; 10 items)* | | | | | | | | | | | |
| --- | --- | --- | --- | --- | --- | --- | --- | --- | --- | --- | --- |
|  | Overall Fit Indices | | | | | |  | Comparative Fit Indices | | | |
|  | χ^2^ | *df* | CFI | TLI | RMSEA | Model Comparison | | | ΔCFI | ΔTLI | ΔRMSEA |
| 1. configural | 1604.27 | 204 | .978 | .971 | .102 (.097, .107) | 1 vs 2 | | | .000 | .005 | -.010 |
| 2. metric | 1613.38 | 244 | .978 | .976 | .092 (.088, .097) |  |  |  |  |  |  |
| 3. scalar | 2018.20 | 484 | .976 | .986 | .069 (.066, .073) | 2 vs 3 | | | .002 | .010 | -.023 |
| *Ruminative Thoughts Style Questionnaire (4 factors; 15 items)* | | | | | | | | | | | |
|  | Overall Fit Indices | | | | | |  | Comparative Fit Indices | | | |
|  | χ^2^ | *df* | CFI | TLI | RMSEA | Model Comparison | | | ΔCFI | ΔTLI | ΔRMSEA |
| 1. configural | 4874.13 | 504 | .973 | .967 | .115 (.112, .118) | 1 vs 2 | | | .001 | .004 | -.008 |
| 2. metric | 4794.58 | 559 | .974 | .971 | .107 (.105, .110) |  |  |  |  |  |  |
| 3. scalar | 4897.32 | 914 | .976 | .983 | .081 (.079, .084) | 2 vs 3 | | | .002 | .012 | -.026 |
| *Distress Tolerance (1 factor; 15 items)* | | | | | | | | | | | |
|  | Overall Fit Indices | | | | | |  | Comparative Fit Indices | | | |
|  | χ^2^ | *df* | CFI | TLI | RMSEA | Model Comparison | | | ΔCFI | ΔTLI | ΔRMSEA |
| 1. configural | 9775.17 | 540 | .913 | .898 | .161 (.158, .164) | 1 vs 2 | | | .011 | .024 | -.020 |
| 2. metric | 8592.37 | 610 | .924 | .922 | .141 (.138, .143) |  |  |  |  |  |  |
| 3. scalar | 8141.20 | 830 | .931 | .947 | .115 (.113, .118) | 2 vs 3 | | | .007 | .025 | -.026 |
| *Inventory of Depression and Anxiety (1 factor [general depression subscale]; 13 items)* | | | | | | | | | | | |
|  | Overall Fit Indices | | | | | |  | Comparative Fit Indices | | | |
|  | χ^2^ | *df* | CFI | TLI | RMSEA | Model Comparison | | | ΔCFI | ΔTLI | ΔRMSEA |
| 1. configural | 3558.59 | 390 | .961 | .953 | .091 (.088, .094) | 1 vs 2 | | | -.002 | .004 | .004 |
| 2. metric | 3837.56 | 450 | .959 | .957 | .087 (.085, .090) |  |  |  |  |  |  |
| 3. scalar | 3491.90 | 640 | .965 | .974 | .067 (.065, .069) | 2 vs 3 | | | .006 | .017 | -.020 |

*Note*. We relied on the model comparison criteria of ΔRMSEA ≤.015 (increase indicates worse fit; Chen, 2007) and ΔCFI/ΔTFI ≤.01 (decrease indicates worse fit; Cheung & Rensvold, 2002) to test for measurement invariance.

Supplemental Table 2

*Cronbach’s alpha values by country*

|  | PFT | CT | RT | AT | DT | Reappraisal | Suppression | IDAS |
| --- | --- | --- | --- | --- | --- | --- | --- | --- |
| United States | .902 | .904 | .948 | .789 | .935 | .902 | .801 | .912 |
| Canada | .903 | .895 | .939 | .787 | .918 | .901 | .755 | .899 |
| South Africa | .875 | .897 | .924 | .721 | .923 | .870 | .729 | .884 |
| Spain | .892 | .852 | .917 | .713 | .899 | .871 | .767 | .887 |
| Argentina | .884 | .831 | .923 | .730 | .901 | .838 | .775 | .886 |
| Uruguay | .844 | .828 | .961 | .799 | .915 | .817 | .761 | .889 |
| England | .857 | .879 | .940 | .690 | .915 | .872 | .769 | .862 |
| Total | .895 | .890 | .940 | .768 | .926 | .889 | .784 | .902 |

*Note.* PFT= problem focused thoughts, CT= counterfactual thinking, RT= repetitive thoughts, AT= anticipatory thoughts, DT = Distress Tolerance, IDAS= Inventory of Depression and Anxiety Scale.
